# From eco-anxiety to eco-paralysis: A case study on behavioral responses to climate change in healthcare professionals

**DOI:** 10.1016/j.joclim.2025.100585

**Published:** 2025-09-16

**Authors:** Matteo Innocenti, Chiara Comerci, Giulia Dockerty, Giovanni Grassi, Gabriele Santarelli, Chiara Cadeddu

**Affiliations:** a Italian Climate Change Anxiety Association (AIACC), Viale Francesco Redi, 127, 50144 Florence, Italy; b Erasmus School of Health Policy and Management, Erasmus University Rotterdam, Burgemeester Oudlaan 50, 3062 PA Rotterdam, Netherlands

**Keywords:** Eco-Anxiety, Eco-Paralysis, Self-Efficacy, Nature-Based Interventions, Healthcare Professionals, Climate Psychology

## Abstract

**Introduction:**

This case report explores the psychological effects of climate change on healthcare professionals through the experience of a dermatologist suffering from climate-related distress.

**Case report:**

The participant developed severe eco-anxiety that evolved into eco-paralysis, impairing her emotional well-being and professional functioning. Her strong commitment to environmental causes contributed to emotional overload, ecological grief, and feelings of helplessness, exacerbated by limited social support and professional isolation.

**Discussion:**

A personalized therapeutic approach was developed, integrating Cognitive Behavioral Therapy (CBT), Acceptance and Commitment Therapy (ACT), and nature-based interventions such as forest bathing. The therapeutic process was focused on grief processing, increasing self-efficacy, and reconnecting with nature, while psychoeducation supported the reframing of environmental concerns and addressed conflicts between personal values and social norms. These strategies reduced eco-paralysis and fostered renewed professional engagement and advocacy.

**Conclusion:**

This case highlights how integrated, evidence-based psychological interventions can address eco-anxiety and its behavioral consequences in healthcare professionals. Enhancing self-efficacy and cultivating emotional resilience through nature and meaning-centered practices can transform climate-related distress into adaptive engagement. This model may inform future clinical practice and case studies; its effectiveness could be investigated in future research.

## Graphical abstract

**Figure:**
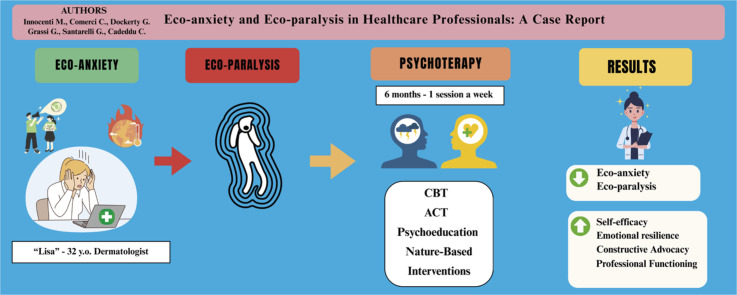

## 1 Introduction

This case report examines the psychological impact of climate change, focusing specifically on a case of eco-anxiety among healthcare professionals. Climate change is recognized as a global crisis [1], leading to an increasing awareness of its effects on mental health [[2], [3], [4]], including eco-anxiety, an emotional response characterized by fear and despair for the future of the environment [[4], [5], [6], [7], [8], [9]]. Some studies suggest that this emotional condition may be relatively common among healthcare students [[10], [11], [12]] highlighting the need to raise awareness while concurrently implementing measures to safeguard their mental health. Evidence underscores the importance of addressing not only the three main ecological crises (climate change, biodiversity loss, and pollution [1], but also climate-related psychological distress to maintain the resilience of healthcare workers [13].

**Fig. 1:**
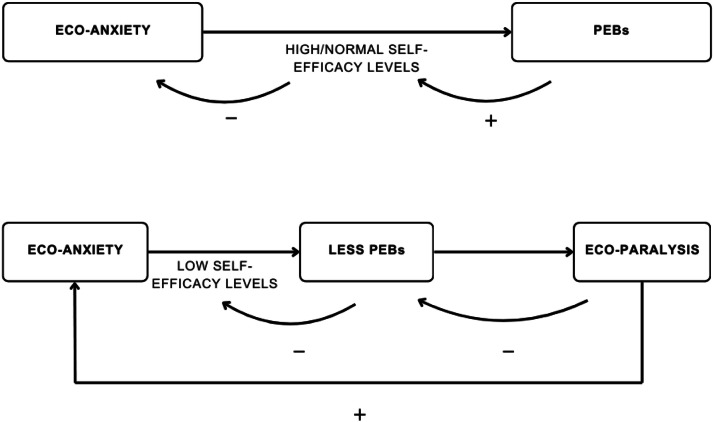
Graphic illustration of the self-efficacy model: eco-anxiety through self-efficacy can stimulate pro-environmental behaviors, which in turn can increase self-efficacy levels and decrease eco-anxiety. On the contrary, when experiencing eco-anxiety, the lack of self-efficacy can lead to engaging in fewer PEBs, leading to eco-paralysis which in turn reduces the sense of self-efficacy and increases eco-anxiety.

The case report explores how eco-anxiety can manifest in complex ways [6,14], influencing attitudes and behaviors while challenging one's sense of purpose and meaning in life. It has been observed [5,[15], [16], [17], [18]] that while eco-anxiety can motivate pro-environmental behaviors, it can also lead to eco-paralysis, where individuals feel powerless to act. This state of inaction can be exacerbated by the perception of helplessness in the face of climate change (Fig. 1).

Within the context of this case study, a treatment plan was developed, and based on previous literature insight [19], an effort was put into formulating a holistic and grief informed model. The model combines Cognitive-Behavioral Therapy (CBT) and Acceptance and Commitment Therapy (ACT) to enhance the participant's self-efficacy [[20], [21], [22]] and coping strategies. More-over, nature-based interventions and tailored encouragements were used to strengthen the connection with the environment and alleviate the grief over its destruction. This documentation of the therapeutic journey is conducted with strict adherence to privacy, with all identifiable information anonymized. Thus, this case report intends to illustrate the critical link between eco-anxiety, mental health, and pro-environmental behaviors, emphasizing the importance of an integrated therapeutic approach.

## 2 Case presentation

This case report delves into the experiences of Lisa, a 32-year-old dermatologist, as a paradigmatic example of how eco-anxiety can evolve into eco-paralysis, particularly among healthcare professionals. Lisa’s story highlights the profound psychological and professional impacts that climate change concerns can exert, especially when intersecting with the demanding nature of healthcare professions.

Lisa has long been deeply committed to environmental causes, a passion that permeates both her personal and professional life. Her lifestyle reflects her convictions: she adheres to a vegan diet, participates in environmental protests, and actively incorporates sustainability practices into her daily routine. At her clinic, she has championed initiatives like reducing single-use plastics, advocating for energy-efficient equipment, and promoting eco-friendly patient education. However, this fervent commitment, while admirable, also left her vulnerable to heightened anxiety regarding climate change, which began to significantly impact her mental health and her capacity to function effectively in her profession.

Initially, Lisa enthusiastically participated in community clean-ups, led recycling campaigns, and worked tirelessly to inspire others to adopt sustainable practices. Yet, over time, she became increasingly disillusioned. Despite her best efforts, she perceived the broader societal and systemic response to climate change as insufficient. She found herself grappling with feelings of frustration and helplessness.

This disillusionment marked the onset of a gradual shift from eco-anxiety to eco-paralysis. Lisa began to feel that her efforts were futile, leading her to question the value of her actions and undermining her motivation. This sense of ineffectiveness not only drained her emotional energy but also began to impair her professional efficacy. As a dermatologist, she struggled to maintain her focus and enthusiasm for patient care, as her eco-anxiety became an all-consuming preoccupation.

As her condition worsened, Lisa turned to avoidance strategies as a means of coping. She began to distance herself from people and situations that heightened her eco-anxiety. Family gatherings became a source of significant distress, as discussions about climate change often led to conflict. Lisa’s family, while acknowledging the reality of climate change, held values and priorities that often clashed with her environmental concerns. These disagreements left her feeling unsupported and alienated, prompting her to avoid such interactions altogether.

The isolation Lisa experienced extended to her professional relationships. She found it increasingly difficult to engage with colleagues who did not share her environmental convictions. This withdrawal further deepened her feelings of despair, as she felt cut off from both her personal and professional support systems. The lack of meaningful connections only compounded her sense of helplessness, creating a vicious cycle of isolation and emotional distress.

Lisa’s experiences were further intensified by the onset of ecological grief—a profound sense of loss for the environment and the future threatened by climate change [23]. She mourned the degradation of ecosystems, the loss of biodiversity, and the irreversible damage inflicted on the natural world. This grief, combined with her eco-anxiety, led Lisa to develop symptoms characteristic of post-traumatic stress disorder (PTSD). These included nightmares, intrusive thoughts, and a persistent sense of dread, significantly impairing her ability to function both personally and professionally. However, these symptoms align more closely with what Kaplan [24] describes as “climate-related pre-traumatic stress disorder (Pre-TSD)”—a condition in which individuals anticipate and emotionally react to future climate-related catastrophes based on their exposure to information about environmental crises.

Lisa’s engagement with detailed accounts of ecological degradation and her pursuit of knowledge about the climate crisis heightened her emotional burden, causing her to pre-emptively grieve for potential future losses. Additionally, some of her distress appeared to stem from “vicarious trauma”—a psychological response triggered by witnessing or learning about the suffering of others, as noted by Pihkala [25]. This phenomenon, common among environmental researchers and advocates, arises from the empathetic engagement with the environmental crisis and the secondary traumatic stress associated with bearing witness to ecological harm.

Adding to this emotional burden was Lisa’s upbringing in a middle-upper socioeconomic family in Northern Italy. Her family’s traditional dietary habits and cultural values often conflicted with her heightened environmental awareness. Her parents, while recognizing the reality of climate change, struggled to integrate its implications into their daily lives. This disconnection left Lisa feeling misunderstood and unsupported, exacerbating her sense of isolation, all aspects already associated in literature with eco-anxiety [26]. The lack of alignment between her personal values and her family’s approach created an internal conflict that further fueled her anxiety and depression. Moreover, the rural origins of her first generation made her both more connected with nature and at the same time field her eco-anxiety [27,28].

As a healthcare professional, she faced unique challenges: the responsibility of caring for others while grappling with her own mental health struggles. The emotional toll of eco-anxiety and ecological grief significantly hindered her ability to provide the compassionate and attentive care her patients deserved. Lisa’s case highlights the urgent need for targeted interventions to address the mental health impacts of climate change, particularly for professionals in high-stress fields like healthcare.

Ultimately, Lisa’s story underscores the importance of recognizing and addressing the psychological dimensions of climate change. By understanding the experiences of individuals like her, we can better identify strategies to prevent eco-anxiety from escalating into eco-paralysis, enabling individuals and communities to work towards a more resilient and sustainable future.

## 3 Method and intervention model

The proposed intervention model incorporates multiple therapeutic strategies aimed at addressing eco-anxiety and its progression into eco-paralysis, particularly for healthcare professionals like Lisa. Essential methods employed include Acceptance and Commitment Therapy (ACT), Cognitive Behavioral Therapy (CBT), enhancing self-efficacy, nature-based interventions, and psychoeducation.

1. **Acceptance and Commitment Therapy (ACT) and Cognitive Behavioral Therapy (CBT):** In Lisa's case, both ACT and CBT [[29], [30], [31], [32]] were implemented to help her manage negative thought patterns and maladaptive behaviors associated with her eco-anxiety. CBT was effective in identifying and altering detrimental thoughts contributing to her feelings of hopelessness and frustration. Conversely, ACT focused on enhancing her psychological flexibility, enabling her to accept her thoughts and feelings about climate change without becoming overwhelmed by anxiety
2. **Enhancing Self-Efficacy:** A core component of the intervention was to increase Lisa's self-efficacy through practical, result-oriented actions [20,33]. She was encouraged to pursue small, tangible outcomes that would bolster her motivation and confidence in making a difference. By leveraging her medical background, Lisa identified specific areas where she could have a meaningful environmental impact, helping her recognize actionable steps aligned with her values. Importantly, she discovered that enhancing her own sense of efficacy also inspired others around her, contributing to collective efficacy. This realization fostered a supportive environment wherein shared efforts toward environmental goals could thrive.
3. **Nature-Based Interventions:** This strategy aimed to deepen Lisa’s connection with the natural world, providing a pathway to effectively manage her eco-anxiety. Activities like spending time outdoors, engaging in community gardening, and practicing forest bathing (Shinrin-Yoku) not only promoted calmness but also increased her appreciation for nature [[34], [35], [36]] These practices played a crucial role in addressing her feelings of existential dread related to ecological grief, helping her recognize the intrinsic value of nature beyond human needs.
4. **Psychoeducation:** Throughout therapy, psychoeducation was vital in equipping Lisa with the knowledge necessary to navigate the complexities of climate change and the psychological impacts of her experience. This approach helped counter misinformation and clarify misconceptions, empowering her to reconcile familial and societal conflicts regarding environmental values [19]. The psychoeducational focus included relational skills, realistic pro-environmental behaviors, and effective communication strategies aimed at bridging understanding with others, thus reducing her feelings of isolation (Table 1).

**Table 1:** Psychological interventions for addressing eco-anxiety and promoting pro-environmental behaviors.

Tool	Process	Purpose
Acceptance and Commitment Therapy (ACT)	Helps individuals accept thoughts and feelings related to eco-anxiety through mindfulness and values-based actions.	Aims to reduce anxiety and foster commitment to pro-environmental behaviors by accepting grief and promoting mental well-being.
Cognitive Behavioral Therapy (CBT)	Identifies and alters negative thought patterns affecting behavior and emotional regulation.	Supports emotional resilience, helping individuals manage eco-anxiety and engage constructively with environmental issues.
Enhancing Self-Efficacy	Involves activities that deepen the connection with nature, such as forest bathing.	Strengthens individual and collective efficacy, transforming anxiety into a motivational force for pro-environmental action.
Nature-Based Interventions	Encourages achieving small, tangible goals to boost motivation and confidence in environmental actions.	Reduces eco-anxiety and ecological grief, fostering a sense of purpose and connection with the environment.
Psychoeducation	Provides information on eco-anxiety and environmental issues to guide realistic goal-setting.	Increases awareness and understanding, empowering individuals to adopt pro-environmental behaviors.

Lisa’s therapy included specific adjustments tailored to her experiences and environmental context [37]. She was guided to reconcile her middle-class upbringing with her ecological principles, understanding that climate change is a symptom of deeper systemic issues. This shift in perspective allowed her to see that individual actions, while significant, are part of broader ecological processes and collective efforts toward sustainability.

Through these methods, the intervention aimed to foster resilience and enhance Lisa's ability to cope with her eco-anxiety and ecological grief, effectively preventing eco-paralysis. By focusing on personal growth, relationship-building, and deeper connections with nature, the intervention model seeks to empower healthcare professionals who confront the psychological ramifications of climate change in both their personal and professional lives [10].

All interventions were conducted by the same therapist, whose clinical orientation integrates established psychotherapeutic methods with a specific sensitivity to climate-related emotional processes. While the therapist’s background is not the central focus of this report, it is relevant to note that their experience includes extensive work on eco-anxiety, environmental emotions, and nature-based therapeutic practices. A more detailed account of this perspective will be provided in a future dedicated publication.

## 4 Results and discussion

In Lisa’s case, the combination of therapeutic interventions effectively reduced paralyzing anxiety and fostered constructive engagement with climate issues. Acceptance and Commitment Therapy (ACT) and Cognitive Behavioral Therapy (CBT) enabled Lisa to reshape her emotional responses and accept the realities of environmental decline, allowing her to address her ecological grief—the profound sadness stemming from the loss of natural environments. By processing this grief, she ultimately alleviated her eco-anxiety.

Enhancing self-efficacy was vital for increasing Lisa's participation in pro-environmental behaviors (PEBs). She engaged with her community through research on the impact of climate change on skin health and by sharing her findings through outreach programs. Nature-based interventions, such as forest bathing, facilitated her reconnection with the natural world, helping her cultivate a deep sense of love and appreciation for the environment. This emotional connection shifted her motivation from fear to a desire to protect nature and its inhabitants.

As her ecological grief lessened, Lisa experienced a significant reduction in eco-anxiety, contributing to decreased feelings of eco-paralysis.

## 5 Conclusion

This study illustrates the potential of multifaceted psychological interventions to alleviate eco-paralysis and enhance engagement in pro-environmental behaviors. The findings emphasize the impact of climate change anxiety on behavior and the critical role of self-efficacy in mediating these effects. By enhancing self-efficacy, individuals can transform their anxiety into a motivational force for positive environmental action. This encouragement not only fosters acceptance of grief associated with ecological loss but also highlights the importance of nature, allowing individuals to find purpose in their actions, contributing to their mental well-being and commitment to environmental stewardship.

Practitioners must encourage patients experiencing environmental distress to collaboratively explore eco-anxiety, framing it as a rational response to the current crisis.

By addressing eco-anxiety and ecological grief, we can foster intergenerational healing that transforms these emotions into eco-awareness, community, agency, and compassion [8,9,38,39]. Future research should explore these interventions across diverse populations to validate these findings and refine treatment approaches for eco-paralysis. Emphasizing therapeutic strategies that involve grief and a renewed love for nature will support individuals in coping with the psychological impacts of climate change while encouraging meaningful commitments to sustainability.

## CRediT authorship contribution statement

**Matteo Innocenti:** Writing – review & editing, Writing – original draft, Methodology, Conceptualization. **Chiara Comerci:** Writing – review & editing, Writing – original draft, Visualization, Methodology, Conceptualization. **Giulia Dockerty:** Writing – review & editing, Writing – original draft. **Giovanni Grassi:** Writing – review & editing, Writing – original draft. **Gabriele Santarelli:** Resources, Investigation, Data curation. **Chiara Cadeddu:** Writing – review & editing, Supervision, Project administration.

## Declaration of competing interest

The authors declare the following financial interests/personal relationships which may be considered as potential competing interests:

Chiara Cadeddu reports article publishing charges was provided by Erasmus Universiteit Rotterdam Erasmus School of Health Policy and Management. Chiara Cadeddu reports a relationship with Erasmus Universiteit Rotterdam Erasmus School of Health Policy and Management that includes: employment.
